# Bettenkapazitätsanalyse für eine internistische Intensivstation: Retrospektive Analyse und Projektion des Intensivbettenbedarfs bei über 65-Jährigen mit ausgewählten kardiovaskulären Erkrankungen

**DOI:** 10.1007/s00063-020-00663-6

**Published:** 2020-02-18

**Authors:** J. S. Radtke, J. Götz, S. Gielen, F. Fischer

**Affiliations:** 1 Fakultät für Gesundheitswissenschaften, Universität Bielefeld, Universitätsstraße 25, 33615 Bielefeld, Deutschland; 2 Klinikum Lippe-Detmold, Detmold, Deutschland

**Keywords:** Intensivmedizin, Demographische Alterung, Kardiovaskuläre Diagnosen, Intensivbettenplanung, Warteschlangentheorie, Intensive care medicine, Demographic aging, Cardiovascular diseases, Intensive care bed planning, Queuing theory

## Abstract

**Hintergrund:**

Der Anstieg der älteren Bevölkerungsgruppe und der damit einhergehende Zuwachs des intensivmedizinischen Bedarfs betont die Notwendigkeit einer effizienten Bettenkapazitätsanalyse. Insbesondere Herz-Kreislauf-Erkrankungen stellen ein häufig auftretendes Erkrankungsbild bei über 65-Jährigen dar. Ziel dieser Arbeit war somit die Analyse des retrospektiven und zukünftigen Intensivbedarfs von älteren Patienten über 65 Jahre mit 6 ausgewählten (kardiovaskulären) Codes der Internationalen statistischen Klassifikation der Krankheiten und verwandter Gesundheitsprobleme (ICD-10) am Beispiel einer Einrichtung der Maximalversorgung in einer ländlichen Region.

**Methodik:**

Für die retrospektive Analyse wurden Daten für den Zeitraum 2015–2017 deskriptiv und bivariat ausgewertet. Die Analyse des Intensivbettenbedarfs erfolgte anhand der Warteschlangentheorie.

**Ergebnisse:**

In dem betrachteten Zeitraum lagen die monatlichen Auslastungsraten kontinuierlich über der idealen Auslastungsrate von 80 % und zum Teil sogar über 100 %. Insbesondere die Nachfrage von Patienten mit I50.14 war im gesamten Krankenhaus sehr hoch. Die Bettenbedarfsanalyse zeigt einen Anstieg von 9 benötigten Betten im Jahr 2017 auf 11 Betten bis zum Jahr 2030 für die 6 Diagnosegruppen. Ohne Einschluss der Diagnosegruppe I50.14 wird sowohl retrospektiv als auch zukünftig etwa die Hälfte der Betten, die bei Einschluss aller 6 Diagnosen benötigt wurden, nachgefragt.

**Diskussion:**

Der Effekt des demographischen Wandels auf den Intensivbettenbedarf ist bereits heute sichtbar. Zudem zeigen die Analysen, dass ein weiterer Anstieg des Bedarfs in Zukunft zu erwarten ist. Die Ergebnisse bestätigen die Notwendigkeit einer an den Bedarf angepassten Intensivkapazitätsplanung. Vor Erweiterung der Bettenkapazitäten wäre jedoch die Analyse von Kriterien, die eine intensivmedizinische Behandlung bedingen, notwendig, um primär Kapazitäten für Patienten mit einem realen Intensivbedarf vorzuhalten.

## Abstract (en)

**Background:**

The increasing number of elderly individuals in the population and the simultaneous increase of the intensive care demand emphasizes the relevance of an efficient bed capacity analysis. Particularly, cardiovascular diseases represent a frequently occurring disease in the population group over 65 years of age. The objective of the following paper is the analysis of the retrospective and prospective intensive care demand by patients over 65 years with 6 selected (cardiovascular) codes of the International Statistical Classification of Diseases and Related Health Problems (ICD-10).

**Methods:**

For the retrospective analysis, data from 2015–2017 were analyzed applying descriptive and bivariate methods. The analysis of the intensive care bed demand was based on the queuing theory.

**Results:**

The monthly capacity utilization rates were constantly higher than the target capacity utilization rate of a maximum of 80% and in some cases even higher than 100%. In particular, the demand of patients with I50.14 was very high throughout the entire hospital. The bed demand analysis shows an increase from 9 needed beds in 2017 to 11 beds by 2030 for the 6 diagnosis groups. Regarding the 5 diagnosis groups without I50.14, only approximately half of the required beds were needed, retrospectively and in future.

**Conclusion:**

The effect of demographic change on the intensive care demand already exists, and a continuing, prospective increase of the demand is expected. The results underline the need of effective and demand-oriented intensive care capacity planning. However, prior to expanding bed capacities, the analysis of admission criteria of intensive care unit patients is necessary to reserve capacities primarily for patients with real intensive care needs.

## Hintergrund

Die Intensivstation (ITS) gehört aufgrund des Einsatzes von lebenserhaltenden sowie -rettenden Maßnahmen für die Behandlung von schwerkranken Patienten zu den kostenintensivsten Bereichen im Krankenhauswesen und bedarf daher einer effizienten Steuerung [1, 8]. Vor dem Hintergrund des demographischen Wandels mit einem Anstieg der älteren Bevölkerung wird in den kommenden Jahren ein Zuwachs des intensivmedizinischen Bettenbedarfs erwartet [12]. Zwischen 2006 und 2016 ist die gesamte Anzahl der Belegungstage auf deutschen ITS bereits um ca. 19 % angestiegen [13]. Insbesondere Herz-Kreislauf-Erkrankungen stellen eine häufig auftretende Indikation bei über 65-Jährigen dar. Aufgrund der gleichzeitig vorliegenden Schwere der teilweise kritischen Erkrankungsbilder (z. B. Myokardinfarkte) sind spezielle intensivmedizinische Therapien erforderlich, sodass internistische ITS mit einem hohen Anteil an älteren Personen ausgelastet sind [5, 9]. Somit ist eine Analyse des intensivmedizinischen Bedarfs der Patientengruppe über 65 Jahren mit kardiovaskulären Erkrankungen notwendig, um die Ressourcen auf der ITS langfristig effektiv einsetzen zu können. Weiterhin könnte sich eine unpräzise Planung und Bereitstellung von Kapazitäten einerseits lebensgefährdend auf die jeweils betroffenen Patienten auswirken und andererseits vermeidbare und hohe Kosten verursachen [1]. Dies verdeutlicht die Notwendigkeit einer effizienten Bettenkapazitätsplanung, u. a. angepasst an den demographischen Alterungsprozess, auf der ITS [11, 14]. Vergangene Studien haben sich bereits mit unterschiedlichen Methoden für eine angemessene Bettenbedarfsberechnung für ITS auseinandergesetzt. Bisher hat sich jedoch kein standardisiertes Verfahren durchgesetzt [8]. Insbesondere mathematische Modelle (z. B. Warteschlangentheorien, computerbasierte Simulationen) eignen sich für eine effiziente Bettenkapazitätsplanung. Aufgrund der Komplexität in der Umsetzung und Anwendung der teilweise sehr anspruchsvollen mathematischen Simulationsmodelle sind diese in der Praxis bislang jedoch kaum verbreitet [17].

Die vorliegende Arbeit stützt sich auf eine Empfehlung von Cochran und Roche [3], die eine simplere Abwandlung der Warteschlangentheorie mithilfe der Software Excel (Microsoft, Redmond, WA, USA) entwickelt haben, um eine barrierefreie Anwendung in der Praxis zu ermöglichen. Ziel der Arbeit ist die Analyse des retrospektiven und zukünftigen Intensivbettenbedarfs mit besonderem Fokus auf Patienten über 65 Jahre mit ausgewählten (kardiovaskulären) ICD-10-Codes am Beispiel eines Maximalversorgers in einer ländlichen Region in Nordrhein-Westfalen.

## Methodik

### Studiendesign

Es wurden Abrechnungsdaten der internistischen ITS eines Maximalversorgers für den Zeitraum Januar 2015 bis Dezember 2017 ausgewertet. In die Analyse wurden die 6 häufigsten Hauptdiagnosen (HD) basierend auf ICD-10-Codes der internistischen ITS miteinbezogen. Von diesen 6 Hauptdiagnosen müssen nach Aussage des Krankenhauses die folgenden zwingend in den ersten 48 h auf der ITS behandelt werden: I21.0 (akuter transmuraler Myokardinfarkt der Vorderwand), I21.1 (akuter transmuraler Myokardinfarkt der Hinterwand), I21.4 (akuter subendokardialer Myokardinfarkt). Außerdem sollen Patienten mit den Diagnosen I49.0 (Kammerflattern und Kammerflimmern) sowie I50.14 (Herzinsuffizienz mit Beschwerden in Ruhe) ebenfalls in den ersten 24 h auf der ITS behandelt werden.

### Datenanalyse – deskriptive Analysen

Die deskriptiven Analysen zur Berechnung der Kapazitätsauslastungen, der Aufnahmeraten sowie der Verweildauern wurden mithilfe der Statistiksoftware IBM SPSS (Version 25; IBM, Armonk, NY, USA) durchgeführt. Für die Berechnung der täglichen Kapazitätsauslastungen (in %) wurde die Anzahl der Patienten um Mitternacht durch die maximale Intensivbettenanzahl von 12 Betten dividiert (Gl. 1). Hieraus ließen sich anschließend die monatlichen (durchschnittlichen) Auslastungsraten berechnen.

1 $$\text{Auslastung/Tag}_\%=\frac{\text{Anzahl Patienten}_{\text{Mitternacht}}}{12\,\text{Betten}}\times 100$$

Die Aufnahmeraten definierten sich über die durchschnittliche Anzahl an Aufnahmen pro Tag je Monat und wurden anhand der folgenden Gl. 2 berechnet:

2 $$AR_{i}=\frac{F_{i,T}}{t_{i,T}}\forall i$$

AR_*i*_ :=: Aufnahmerate für Station *i* pro Tag
*F*_*i,T*_ :=: Fälle auf Station *i* in Zeitraum *T*
*t*_*i,T*_ :=: Anzahl Tage auf Station *i* im Zeitraum *T*

Um die durchschnittliche Verweildauer pro Monat zu ermitteln, wurde die Verweildauer jedes einzelnen Falls anteilig auf jeden Monat aufgeteilt. Anschließend wurde die Summe der Verweildauertage jedes Monats durch die Anzahl der Patienten in dem jeweiligen Monat dividiert, um die durchschnittliche Verweildauer pro Fall zu erfahren. Hierfür wurde Gl. 3 verwendet:

3 $$VD_{i,T}=\frac{\begin{array}{c}\text{Anzahl Tage aller Patien-}\\\text{ten im Zeitraum T}\end{array}}{\begin{array}{c} \text{Anzahl Patienten}\\ \text{im Zeitraum T}\end{array}}\forall i$$

$$VD_{i,T}:=$$
Verweildauer auf Station *i* im Zeitraum *T*

### Bettenbedarfsanalyse für Patienten über 65 Jahre mit ausgewählten ICD-10-Codes

Die hier angewandte Bettenbedarfsanalyse für die Patientengruppe der über 65-Jährigen mit den 6 ausgewählten HD wurde basierend auf der Warteschlangentheorie durchgeführt und orientiert sich an der Studie von Cochran und Roche [3], die sich wiederum an dem M/M/c-Modell des Warteschlangensystems zur Bettenbedarfsberechnung orientiert^1^. Die Warteschlangentheorie (engl.: „queuing theory“) stellt ein mathematisches Vorgehen dar, mithilfe dessen eine effiziente Verteilung von limitiert vorhandenen Ressourcen (*Intensivbetten*) ermittelt wird. Ein Dienstleistungszentrum (*ITS*) kann eine bestimmte Anzahl von Kunden (*Intensivpatienten*), die den Service (*Intensivbehandlung*) benötigen, bis zu einer bestimmten Anzahl simultan bedienen. Sobald die vorhandenen Kapazitäten (*Intensivbetten*) ausgelastet sind, muss ein neuer Kunde (*Intensivpatient*) warten [2].

Das Verfahren wurde in dieser Arbeit mithilfe von Microsoft Excel 2016 verwendet. Für die Berechnung des Bettenbedarfs, beruhend auf der Warteschlangentheorie, waren die Parameter Bettenkapazitäten, Verweildauer und Aufnahmeraten grundlegend. Für die Intensivpatienten wurde die berechnete Verweildauer miteinbezogen und für die Normalstationspatienten (mit einer der ausgewählten HD) ist die Annahme getroffen worden, dass diese eine durchschnittliche Verweildauer von 2 Tagen auf der ITS aufweisen würden. Aufgrund der hohen Anzahl an Patienten mit dem ICD-10-Code I50.14 auf der ITS (Abb. 2) und der noch bedeutsameren Patientenzahl auf der Normalstation (2015: 376 Patienten, 2016: 429 Patienten, 2017: 387 Patienten) ist davon auszugehen, dass der Einschluss dieser Patientengruppe bei der Bettenbedarfsberechnung eine Verzerrung des eigentlichen Intensivbettenbedarfs hervorrufen könnte. Da retrospektiv nicht einzuschätzen ist, inwieweit ein tatsächlicher Bedarf an Intensivplätzen bei der hohen Anzahl an Normalstationspatienten mit diesem ICD-10-Code vorlag, wurde die Bettenbedarfsberechnung sowohl für die gesamte Patientengruppe mit allen 6 HD als auch für die Gruppe ohne Einschluss des ICD-10-Codes I50.14 durchgeführt. Der Bettenbedarf für die beiden Gruppen ist mit den folgenden Gln. 4 und 5 berechnet worden:

4 $$B_{T}=\sum _{i}AR_{i,T}* VD_{i,T}$$

$$B_{T}:=$$
Bettenbedarf für Zeitraum T (bei einer Zielauslastung von 100 %)
$$\mathrm{AR}_{i,T}:=$$
Aufnahmerate für Station *i* pro Tag für Zeitraum *T*
$$\mathrm{VD}_{i,T}:=$$
Verweildauer auf Station *i* in Tagen für Zeitraum *T*

5 $$C=\frac{B}{\rho }$$

$$\mathrm{C}:=$$
Bettenbedarf (bei einer Zielauslastung von 80 %)
$$\mathrm{B}:=$$
Bettenbedarf (bei einer Zielauslastung von 100 %)
$$\uprho :=$$
Zielauslastung (80 %)

Um anschließend den Bettenbedarf für diese Patientengruppe für 2020, 2025 und 2030 zu prognostizieren, wurde der Bettenbedarf mithilfe des prozentualen Bevölkerungsgruppenanstiegs für die 3 Zeiträume aus der Bevölkerungsvorausberechnung für die Region berechnet. Für die Prognoserechnung wurde die Annahme getroffen, dass sowohl die Verweildauer als auch die Zielauslastungsrate konstant bleibt. Für die Zielauslastung wurden 80 % festgelegt, um die Möglichkeit zu haben, jederzeit intensivpflichtige Notfälle aufnehmen und zudem kurzfristigen Personalausfall (z. B. durch Erkrankungen) auffangen zu können.

**Figure 1:**
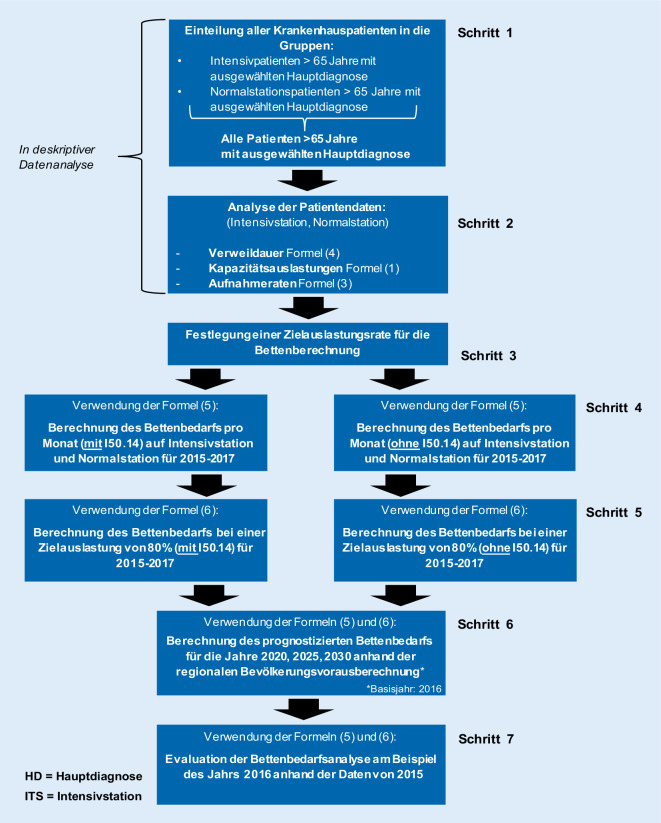

Die Bedarfsberechnung für 2016 diente als Basis für die Hochrechnungen, da für 2017 noch keine Informationen über die Bevölkerung (und insbesondere den Bevölkerungsanteil der über 65-Jährigen) in der Region zur Verfügung gestanden haben. Zunächst war die Berechnung der Aufnahmerate für diese Patientengruppe in den Jahren 2020, 2025 und 2030 wichtig, bevor anschließend der Bettenbedarf für diese Jahre prognostiziert wurde. Für die Berechnung der zukünftigen Aufnahmerate wurde Gl. 2 entsprechend mit dem Wachstumsfaktor des jeweiligen Jahrs multipliziert. Anschließend wurde die neu berechnete Aufnahmerate in die Gln. 4 und 5 eingesetzt, um den Bettenbedarf für das individuelle Jahr zu berechnen. Die Vorgehensweise der Bettenbedarfsanalyse ist in Abb. 1 detailliert dargestellt.

## Ergebnisse

### Deskriptive Analysen

**Figure 2:**
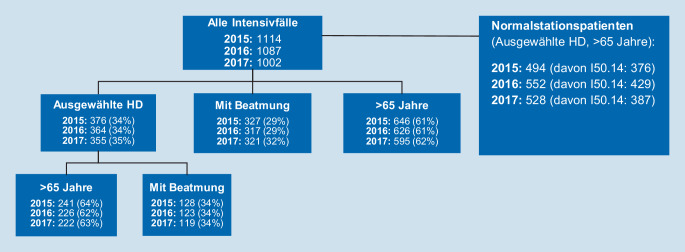

In allen 3 betrachteten Jahren (2015–2017) waren jeweils etwas mehr als 1000 Patienten auf der ITS. Das durchschnittliche Alter der Intensivpatienten lag im Jahr 2015 bei 66,7 Jahren, 2016 bei 67,4 Jahren und 2017 bei 66,6 Jahren. Bei ungefähr einem Drittel der Intensivpatienten in den Jahren 2015–2017 wurde eine der in dieser Studie fokussierten Hauptdiagnosen diagnostiziert. Innerhalb dieser zusammengefassten Diagnosegruppen waren ca. zwei Drittel der Patienten über 65 Jahre alt. Weiterhin mussten 34 % der Intensivpatienten mit den untersuchten Hauptdiagnosen beatmet werden. Die Intensivfälle mit Beatmungspflicht sind zwischen 2015 und 2017 von 29 % auf 32 % leicht angestiegen. Der Anteil der über 65-jährigen Intensivpatienten war in den 3 Jahren relativ stabil. Zusätzlichen wurde in jedem Jahr ein hoher Anteil (mind. ein Drittel der gesamten Intensivpatienten) an Patienten die älter als 65 Jahre sind und eine der ausgewählten Hauptdiagnosen hatten, lediglich auf der Normalstation behandelt (Abb. 2).

Insgesamt zeigt sich in allen 3 betrachteten Jahren eine deutliche Überauslastung (Tab. 1). Der Anteil der Tage mit einer Unterauslastung (unter 70 %) ist in den Jahren sehr gering und hat sich zwischen 2015 und 2017 deutlich reduziert. Obwohl sich der Anteil der Tage mit einer idealen Auslastung von 2015 (1,6 %) bis 2016 und 2017 (jeweils 3 %) fast verdoppelt hat, ist eine ideale Auslastung dennoch sehr selten. Während sich der Anteil der Tage mit einer Überauslastung zwischen 80 und 100 % zwischen 2015 (78,6 %), 2016 (68,9 %) und 2017 (52,6 %) reduzierte, stieg gleichzeitig der Anteil der Tage mit einer starken Überauslastung (über 100 %) innerhalb der 3 Jahre deutlich an. Im Jahr 2017 hatten demnach mehr als doppelt so viele Tage wie im Jahr 2015 eine Auslastung von über 100 % (161 vs. 64 Tage).

**Table 1:** 

	Tage	2015	2016	2017
*Unterauslastung* *(0–69,99* *%)*	Anzahl (%)	8 (2,2)	2 (0,5)	1 (0,3)
*Ideale Auslastung* *(70,00–80,00* *%)*	Anzahl (%)	6 (1,6)	11 (3,0)	11 (3,0)
*Überauslastung* *(80,01–100,00* *%)*	Anzahl (%)	278 (78,6)	252 (68,9)	192 (52,6)
*Starke Überauslastung* *(>100,00* *%)*	Anzahl (%)	64 (17,5)	101 (27,6)	161 (44,1)
*Insgesamt*	Anzahl (%)	365 (100)	366 (100)	365 (100)

Anhand der monatlichen Aufnahmeraten lassen sich keine saisonalen Besonderheiten identifizieren (Tab. 2). So weist jedes Jahr einen individuellen Verlauf der Aufnahmen auf. Die meisten Aufnahmen auf der ITS gab es für das Jahr 2015 in den Monaten Januar und Februar mit durchschnittlich je 3,8 Aufnahmen pro Tag, für das Jahr 2016 in dem Monat April (3,9 Aufnahmen pro Tag) und für das Jahr 2017 in dem Monat September (3,3 Aufnahmen pro Tag). In Bezug auf die Aufnahmeraten lässt sich feststellen, dass die meisten aufgenommenen Intensivpatienten im Durchschnitt älter als 65 Jahre waren. Weiterhin wurde im Durchschnitt täglich mindestens ein Patient mit einer der ausgewählten Hauptdiagnosen auf der ITS aufgenommen. Bei durchschnittlich 3 Gesamtaufnahmen pro Tag auf der ITS wird die Bedeutung des Anteils von Patienten dieser Diagnosegruppen somit ersichtlich. Betrachtet man weiterhin die täglichen Aufnahmeraten von Patienten über 65 Jahre mit einer der ausgewählten HD, ergibt sich für diese Gruppe ebenfalls ein bedeutender Anteil an der gesamten Intensivpatientenzahl. Die durchschnittlichen Aufnahmeraten von Beatmungspatienten sind für die Jahre 2015, 2016 und 2017 identisch. Demnach wurde durchschnittlich ein Patient mit Beatmungspflicht täglich auf der ITS aufgenommen.

**Table 2:** 

Monat	Aufnahmen	Alle ITS-Patienten			Patienten >65 Jahre			Patienten mit untersuchten HD			Patienten mit untersuchten HD und >65 Jahre			Beatmungspatienten		
		2015	2016	2017	2015	2016	2017	2015	2016	2017	2015	2016	2017	2015	2016	2017
Jan	*Pro Tag*	3,8	3,6	2,7	2,6	2,4	1,8	1,4	0,9	1,1	1,0	0,6	0,7	1,3	0,9	1,5
Feb	*Pro Tag*	3,8	3,6	3,1	2,3	2,1	2,3	1,4	1,4	0,9	1,0	0,9	0,6	1,4	1,4	1,1
März	*Pro Tag*	2,5	3,5	2,6	1,5	2,3	1,7	0,7	1,1	0,9	0,5	0,8	0,5	1,0	1,3	1,2
Apr	*Pro Tag*	2,7	3,9	3,1	1,6	2,2	2,0	1,1	1,1	1,3	0,6	0,7	0,8	0,9	1,2	1,2
Mai	*Pro Tag*	2,9	2,7	2,7	1,6	1,6	1,5	1,4	1,3	1,1	0,8	0,7	0,6	1,0	0,9	1,0
Juni	*Pro Tag*	2,8	2,4	2,8	1,8	1,5	1,5	1,1	1,0	1,1	0,8	0,6	0,5	0,7	0,7	0,9
Juli	*Pro Tag*	3,3	3,0	2,8	1,8	1,7	1,8	1,0	1,2	1,2	0,5	0,7	0,7	0,9	1,0	0,8
Aug	*Pro Tag*	3,6	2,9	2,5	2,0	1,5	1,5	1,0	0,9	0,7	0,6	0,5	0,5	1,2	0,8	0,6
Sept	*Pro Tag*	3,7	2,7	3,3	2,0	1,6	2,0	1,1	1,0	1,2	0,6	0,7	1,0	0,8	0,9	1,0
Okt	*Pro Tag*	3,1	3,7	2,9	2,0	2,0	1,8	1,2	1,0	1,2	0,9	0,5	0,7	1,0	0,7	1,0
Nov	*Pro Tag*	3,0	3,4	3,0	1,7	1,9	1,7	1,1	1,3	0,9	0,8	0,7	0,6	0,7	1,0	0,6
Dez	*Pro Tag*	3,2	2,5	3,3	1,3	1,2	1,3	0,7	0,6	0,9	0,4	0,5	0,5	0,6	0,5	0,6
**Insgesamt**	***Pro Tag***	**3,2**	**3,2**	**2,9**	**1,9**	**1,8**	**1,7**	**1,1**	**1,1**	**1,0**	**0,7**	**0,7**	**0,7**	**1,0**	**1,0**	**1,0**

Die durchschnittliche Verweildauer pro Patient ist zwischen 2015 und 2017 um ca. 10 % angestiegen: Im Jahr 2015 lagen Patienten im Durchschnitt 4,2 Tage auf der ITS, im Jahr 2016 waren es 4,3 Tage und 2017 waren es 4,6 Tage. In allen 3 Jahren ist erkennbar, dass Beatmungspatienten im Durchschnitt am längsten (2015: 7,2 Tage; 2016: 7,0 Tage; 2017: 7,5 Tage) auf der ITS lagen. Die anderen 3 Untergruppen zeigten hingegen relativ ähnliche Verweildauern zwischen 4 und 5 Tagen auf (Tab. 3).

**Table 3:** 

Monat	Verweildauer	Alle ITS-Patienten			Patienten >65 Jahre			Patienten mit untersuchten HD			Patienten mit untersuchten HD und >65 Jahre			Beatmungspatienten		
		2015	2016	2017	2015	2016	2017	2015	2016	2017	2015	2016	2017	2015	2016	2017
Jan	*In Tagen*	3,6	3,9	5,3	3,9	8,3	4,1	3,5	4,6	3,1	3,8	5,7	3,3	5,9	5,4	6,0
Feb	*In Tagen*	3,9	4,2	4,8	4,0	3,9	4,9	3,5	3,2	4,2	3,4	3,0	3,7	6,1	6,7	8,6
März	*In Tagen*	4,9	4,3	5,1	5,4	4,1	4,8	5,4	4,9	5,3	6,2	5,5	6,1	8,0	6,7	7,2
Apr	*In Tagen*	4,5	3,7	4,6	4,9	4,2	5,0	5,4	3,7	5,5	6,1	4,2	6,1	8,7	5,4	7,8
Mai	*In Tagen*	4,4	4,6	4,9	4,3	4,9	4,9	5,0	4,9	5,1	4,2	6,4	4,7	7,7	6,2	7,4
Juni	*In Tagen*	4,6	5,1	4,5	4,2	5,7	5,0	4,8	6,5	5,2	2,8	7,8	6,1	8,4	8,7	8,2
Juli	*In Tagen*	4,1	4,4	4,5	4,3	5,1	4,1	3,2	5,9	4,7	3,3	7,5	5,5	6,8	7,6	8,1
Aug	*In Tagen*	3,8	4,4	4,8	3,8	5,6	5,0	4,4	5,3	6,7	3,3	6,7	7,0	5,2	9,5	8,4
Sept	*In Tagen*	3,7	4,6	3,9	3,7	4,8	3,5	5,2	4,1	3,9	4,9	3,9	3,7	7,0	8,2	5,8
Okt	*In Tagen*	4,3	3,6	4,5	3,5	3,6	3,8	5,2	4,3	5,2	4,0	3,3	3,8	7,8	5,5	7,4
Nov	*In Tagen*	4,3	3,9	4,3	4,4	4,0	3,6	4,6	4,5	3,6	4,5	3,7	2,9	9,1	6,6	8,6
Dez	*In Tagen*	3,9	5,0	4,6	3,7	4,0	3,4	2,5	5,0	4,2	2,5	4,8	3,6	5,9	6,9	6,2
**Insgesamt**	***In Tagen***	**4,2**	**4,3**	**4,6**	**4,2**	**4,9**	**4,4**	**4,4**	**4,7**	**4,7**	**4,1**	**5,2**	**4,7**	**7,2**	**7,0**	**7,5**

### Retrospektive Bettenbedarfsanalyse für Patienten über 65 Jahre mit ausgewählten ICD-10-Codes

In Tab. 4 ist der Bettenbedarf für Patienten über 65 Jahre mit den ausgewählten HD für jedes Jahr dargestellt. Die Tabelle stellt den durchschnittlichen Bettenbedarf bei einer Zielauslastungsrate von 80 % sowohl seitens der Patienten mit den ausgewählten HD, die auf der ITS behandelt wurden, als auch derjenigen Patienten, die auf der Normalstation behandelt wurden, dar. Während die jeweils linken Spalten alle 6 HD inkludieren, beruhen die Werte aus den jeweils rechten Spalten auf den 5 HD ohne Einschluss von I50.14. Demnach hätte man im Jahr 2015 durchschnittlich 8 Betten (I1 + N1) benötigt, um der Nachfrage dieser Patientengruppe mit allen 6 HD gerecht zu werden und um gleichzeitig die Zielauslastungsrate von 80 % einzuhalten. Es lässt sich erkennen, dass Patienten mit der HD I50.14 mehr als die Hälfte der nachgefragten Betten (4 Betten) von allen 6 HD (7,7 Betten) im Jahr 2015 in Anspruch nahmen. Somit wurden von den Patienten in der dargestellten Altersgruppe mit den verbliebenen HD insgesamt (aufgerundet) 4 Betten im Durchschnitt pro Tag im Jahr 2015 benötigt. Im Jahr 2016 ist ein Anstieg des Bettenbedarfs zu verzeichnen, da in diesem Jahr durchschnittlich 9 Betten pro Tag für Patienten über 65 Jahre mit den ausgewählten 6 HD benötigt wurden. Hingegen blieb der Bettenbedarf für die analysierte Patientengruppe ohne I50.14 konstant. Der durchschnittliche Bettenbedarf für die Patientengruppe (mit I50.14) änderte sich im Jahr 2017 nicht und verblieb somit bei einer unveränderten Anzahl von (aufgerundet) 9 Betten pro Tag. Ebenso blieb der Wert des Bettenbedarfs für die Gruppe ohne Berücksichtigung von I50.14 – wie in den beiden Jahren davor – mit (aufgerundet) 4 Betten konstant.

**Table 4:** 

2015		2016		2017	
Bettenbedarf *(mit I50.14)*	Bettenbedarf *(ohne I50.14)*	Bettenbedarf *(mit I50.14)*	Bettenbedarf *(ohne I50.14)*	Bettenbedarf *(mit I50.14)*	Bettenbedarf *(ohne I50.14)*
8 (7,7)	4 (3,7)	9 (8,9)	4 (4,0)	9 (8,5)	4 (4,0)

^a^Aufgrund der Aufrundung der Werte auf die erste Nachkommastelle kann die dargestellte Summe von der Summe der Einzelwerte abweichen

### Prognostizierte Bettenbedarfsanalyse für Patienten über 65 Jahre mit ausgewählten ICD-10-Codes

**Figure 3:**
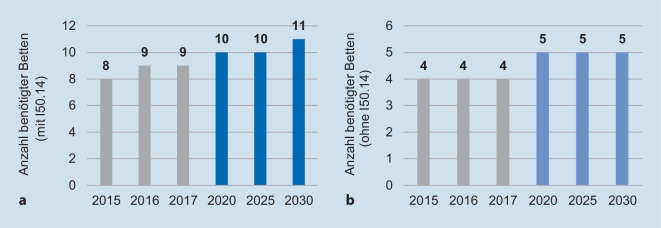

Die Abb. 3 bildet die prognostizierte Vorausberechnung des Bettenbedarfs einmal für die gesamten betrachteten HD-Gruppen (mit I50.14) als auch nur für die 5 HD-Gruppen (ohne I50.14) der über 65-Jährigen für die Jahre 2020, 2025 und 2030 ab. Unter Einschluss aller 6 ICD-10-Codes lässt sich erkennen, dass der Bettenbedarf bis 2030 um insgesamt 2 Betten ansteigen wird. Aufgrund des Anstiegs der älteren Bevölkerung (über 65 Jahre) in der Region um ca. 0,9 % zwischen 2016 und 2020 wird sich der Bettenbedarf um ein Bett auf (aufgerundet) 10 (9,5) Betten bis zum Jahr 2020 erhöhen. Bis 2025 steigt der Bedarf nur geringfügig auf 9,6 Betten – trotz Wachstum der Bevölkerungsgruppe um 6,6 % – an. Aufgrund des Anstiegs der Altersgruppe über 65 Jahre in der Region um ca. 7,1 %, wird erwartet, dass die betrachtete Patientengruppe (über 65 Jahre mit einer der 6 HD) im Jahr 2030 voraussichtlich 11 Betten nachfragen wird (Abb. 3a).

Bei Ausschluss des ICD-10-Codes I50.14 lässt sich eine leicht veränderte Entwicklung darstellen. Der Bettenbedarf der 5 HD blieb für alle 3 Jahre (2015–2017) konstant bei durchschnittlich 4 Betten. Ohne Einschuss des ICD-10-Codes I50.14 ist der Anstieg des Bettenbedarfs etwas langsamer (Abb. 3b).

Das Bevölkerungsgruppenwachstum der Einwohner über 65 Jahre in der Region (+0,9 %) zwischen 2016 und 2020 wird einen sichtbaren Einfluss auf die Bettennachfrage haben. Demnach wird im Jahr 2020 ein Bettenbedarf von aufgerundet 5 Betten (4,2 Betten) für die fokussierte Patientengruppe erwartet. Der Bedarf steigt zwar anschließend zwischen 2020 und 2025 bei einem Bevölkerungsgruppenwachstum von 6,6 % noch einmal auf 4,3 Betten und anschließend bis 2030 bei einem relativen Wachstum von 7,1 % auf 4,6 Betten an, jedoch wird die aufgerundete Bettenanzahl von durchschnittlich 5 nachgefragten Betten pro Patient über 65 Jahre mit einer der ausgewählten HD folglich konstant bleiben (Abb. 3b).

### Evaluation der Praktikabilität der prognostizierten Bettenbedarfsanalyse

Um die Praktikabilität der hier angewandten Bettenbedarfsprognose anhand des relativen Bevölkerungswachstums zu überprüfen, wurde basierend auf der Bettenbedarfsberechnung (ohne I50.14) von 2015 der Bettenbedarf für 2016 mit einem Wachstum in der Bevölkerungsgruppe der über 65-Jährigen in der Region von 1,1 % hochgerechnet und mit der tatsächlichen Bettenbedarfsberechnung (ohne I50.14) von 2016 verglichen. Zusammengefasst gibt es keinen Unterschied zwischen dem realen durchschnittlichen Bettenbedarf und dem prognostizierten Bettenbedarf für das Jahr 2016. Beide Berechnungen kommen zu einer Anzahl von durchschnittlich 4 benötigten Betten pro Tag in dem betrachteten Jahr (Ist: 4,0/Prognose: 3,7). Vergleicht man den prognostizierten Bettenbedarf mit dem tatsächlichen Bettenbedarf pro Monat, werden ebenfalls nur geringe Abweichungen (in der Regel ±1 Bett) zwischen den Monaten erkennbar.

## Diskussion

Die im Rahmen dieser Studie gewonnenen Ergebnisse mittels deskriptiver Analysemethoden und der angewendeten Bettenbedarfsberechnung bestätigen die Relevanz einer effizienten und bedarfsgerechten Bettenkapazitätsanalyse. Diese Studie untersuchte unter anderem, wie sich der Intensivbettenbedarf, auch hinsichtlich relevanter Patientengruppen, in dem betrachteten Zeitraum (2015–2017) in einer Einrichtung der Maximalversorgung in einer ländlichen Region darstellte.

Basierend auf der Analyse der Kapazitätsauslastungen ist festzuhalten, dass die primäre Schwierigkeit der internistischen ITS in einer Überauslastung der Intensivbetten zu finden ist. Obwohl das Krankenhaus die von der Deutschen Interdisziplinären Vereinigung für Intensiv- und Notfallmedizin (DIVI) und der European society of intensive medicine (ESICM) maximal empfohlene Intensivbettenanzahl von 12 Betten bereitstellt [7, 16], zeigt sich, dass die Nachfrage höher als das (Betten‑)Angebot ist. Insbesondere die häufig auftretenden Auslastungsraten über 100 %, die in einer Erhöhung der Bettenanzahl resultierten, machen die Notwendigkeit einer effizienten Intensivkapazitätsplanung deutlich. Eine ideale Bettenauslastung sollte aufgrund des Arbeits- und Zeitdrucks und aufgrund der hohen Verantwortung, die das Personal einer Intensivstation zu tragen hat, angestrebt werden, zumal Überauslastungen zu zusätzlichen Arbeitsbelastungen beim Personal führen werden [1, 10]. Dies wiederum könnte zu negativen Folgen in Bezug auf die Patientensicherheit beitragen [1].

Bei der Analyse von zeitlichen Schwankungen in den Aufnahmeraten sowie der Verweildauer lassen sich individuelle Verläufe identifizieren. Hierbei muss berücksichtigt werden, dass die Dokumentationsqualität zu Verzerrungen führen kann und somit die jeweilige Codierung der Haupt- und Nebendiagnosen einen wesentlichen Einfluss auf die diagnoseabhängigen Auslastungsraten einnimmt. Hohe Auslastungsraten können wiederum zu fehleranfälligen Dokumentationen führen. Zu bedenken sind auch mögliche Fehlanreize im Abrechnungsprozess, um die Kostenintensität von ITS zu kompensieren [15]. Somit ermöglichen die Ergebnisse keine eindeutige Extrapolation dahingehend, ob es zu einer bestimmten Jahreszeit mehr oder weniger Aufnahmen gab bzw. der Auslastungsgrad unterschiedlich hoch war.

Ungefähr zwei Drittel der Patienten waren im Alter über 65 Jahre und der prozentuale Anteil hat zwischen 2015 und 2017 bereits zugenommen. Verlässt man sich weiterhin auf die Medikalisierungsthese, die besagt, dass bei steigender Lebenserwartung die Nachfrage nach medizinischen und pflegerischen Leistungen proportional zunehmen wird [4], ist auch zukünftig mit einem vermehrten Bedarf an Intensivbetten für die betrachtete Altersgruppe zu rechnen.

Aufgrund der hohen Anzahl an Fällen mit dem ICD-10-Code I50.14 auf der Normalstation wurden für jedes Jahr 2 Bettenbedarfsanalysen durchgeführt. Die beiden Analysen zeigen auf, welche Konsequenzen in der Intensivbettennachfrage entstehen würden, wenn zukünftig alle Patienten über 65 Jahre mit dem ICD-10-Code I50.14 für mindestens 48 h auf der ITS behandelt werden. Dementsprechend wäre die Hälfte der nachgefragten Betten seitens der 6 betrachteten HD auf die Diagnose I50.14 zurückzuführen. Konsequenterweise sollte erwogen werden, ob die Zielauslastungsrate von 80 % erhöht wird, um nicht unnötige Leerkapazitäten zurückzuhalten. Dies kann jedoch aufgrund volatiler Nachfrage problematisch sein und Wartezeiten auf der ITS sollten unbedingt vermieden werden.

Im Rahmen der Bettenbedarfsprognose für 2020, 2025 und 2030 ist zu erwähnen, dass sich ein Anstieg in der Nachfrage zwischen jedem Jahr ereignete, dieser aber aufgrund der Aufrundung der Bettenanzahl auf ganze Betten nicht sichtbar wird. Eine Aufrundung der Bettenanzahl könnte jedoch in manchen Jahren zu einer Überschätzung des Bedarfs führen (z. B. 2020: 4,2 Betten für alle 5 Diagnosegruppen ohne I50.14). Anhand der Prognoserechnung wird der Einfluss des demographischen Wandels auf den Intensivbedarf und somit die Bedeutsamkeit von Patienten im höheren Alter erkennbar. Insofern eignet sich die hier angewandte Analyse als sinnvolle Methode, um den Intensivbedarf, justiert an den demographischen Wandel, zu projizieren. Bedenkt man, dass die Patientengruppe der über 65-Jährigen mit einer der ausgewählten HD etwas mehr als ein Fünftel von allen Intensivpatienten ausmacht und die ITS nur über 12 Intensivbetten verfügt, ist sowohl der aktuelle als auch der zukünftige Bettenbedarf für die fokussierte Patientengruppe sehr hoch, insbesondere bei Einbezug der Diagnose I50.14. Diese Tatsache betont die Notwendigkeit, die Erkrankungsschwere bei der Aufnahme von Patienten zu ermitteln, um festzustellen, inwieweit eine Intensivpflichtigkeit tatsächlich vorliegt. Außerdem sollte vor einer Ausweitung der Bettenkapazitäten zunächst überprüft werden, inwieweit die eingesetzten Ressourcen und Behandlungsabläufe optimiert werden können, um die vorhandenen Kapazitäten effizienter zu nutzen und Ineffizienzen langfristig zu vermeiden.

## Limitationen

Einige methodische Limitationen sind zu beachten. Zunächst sind die Ergebnisse nur bedingt auf andere Kliniken übertragbar, da nur ein Maximalversorger in die Untersuchung eingegangen ist. Der Bedarf an Intensivbetten ist jedoch stark von den individuellen Strukturen (z. B. hinsichtlich der Kriterien zur Aufnahme auf die ITS oder der Versorgung von I50.14) und regionalen Gegebenheiten abhängig. Um ein noch dezidierteres Bild des Intensivbedarfs älterer Patienten abbilden zu können, sind zudem noch weitere wesentliche Diagnosen, wie z. B. Infektionen und Pneumonien, in die Kapazitätsanalyse einzubeziehen. Das Forschungsinteresse dieser Arbeit zielte aber auf die genannten kardiovaskulären Hauptdiagnosen. Weitere Diagnosen sollten somit in zukünftigen Studien Beachtung finden.

Die prospektive Bedarfsplanung basiert auf der Annahme, dass lediglich Veränderungen in der Altersstruktur zu berücksichtigen sind. Der Bedarf kann sich jedoch auch bedingt durch weitere Faktoren, insbesondere durch den epidemiologischen Wandel und medizinisch-technischen Fortschritt, verändern.

## Fazit für die Praxis

- Die dargestellte ITS weist sehr hohe Auslastungsraten (teilweise über 100 %) auf und bedarf daher einer Umstrukturierung der Intensivbettenplanung.
- Die Berechnung des Intensivbettenbedarfs unterliegt einer schwierigen Herausforderung. Die für die Arbeit herausgearbeitete Methodik, basierend auf der Warteschlangentheorie, hat sich jedoch als effektiv und praktikabel bewährt.
- Die Studie bestätigt die Relevanz der Intensivpatientengruppe über 65 Jahre mit kardiovaskulären Erkrankungen. Ein zukünftiger Effekt des demographischen Wandels auf den ansteigenden intensivmedizinischen Bedarf dieser Patientengruppe konnte abgeleitet werden.
- Vor Erweiterung der Intensivkapazitäten sollten standardisierte Aufnahmekriterien für die ITS bestimmt werden. Die Aufnahme aller Intensivpatienten mit einer bestimmten Diagnose – unabhängig von der Erkrankungsschwere – (z. B. I50.14) würde eine viel zu große und nicht realisierbare Bettenanzahl auf der ITS voraussetzen.

## Footnotes

1 Im M/M/c-Warteschlangensystem sind Ankunftszeit und Bedienzeit (hier: Verweildauer) exponenziell verteilt. Die Wartenden werden von c Agenten (hier: Intensivbetten) bedient. Das Modell nimmt einen unbegrenzten Warteraum an und es gibt keine Einschränkungen hinsichtlich der Wartezeit der Kunden (hier: Intensivpatienten; [6]). Dieses Modell stellt die Basis für die Bettenbedarfsberechnung dar.
